# Detection of Adulterated Oregano Samples Using Untargeted Headspace–Gas Chromatography–Ion Mobility Spectrometry Analysis

**DOI:** 10.3390/foods13040516

**Published:** 2024-02-07

**Authors:** Blas Rocamora-Rivera, Natalia Arroyo-Manzanares, Pilar Viñas

**Affiliations:** Department of Analytical Chemistry, Faculty of Chemistry, University of Murcia, 30100 Murcia, Spain; blas.rocamorar@um.es (B.R.-R.); pilarvi@um.es (P.V.)

**Keywords:** oregano, adulteration, gas chromatography, ion mobility spectrometry, headspace

## Abstract

Oregano is often adulterated for economic reasons. This fraud mainly consists of adding other species with lower commercial value, such as olive leaves. To ensure the authenticity of oregano, an analytical method based on the analysis of the volatile organic compound (VOC) profile obtained by headspace gas chromatography coupled to ion mobility spectrometry (HS-GC-IMS) was developed and validated. Samples of ecological Mediterranean oregano adulterated with different percentages of two types of olive leaves (cornicabra and manzanilla) were studied using a non-targeted analysis. Moreover, a total of 30 VOCs were identified in the analyzed samples, and 24 compounds could be quantified using calibration curves based on Boltzmann’s equation. A chemometric model based on orthogonal partial least squares discriminant analysis (OPLS-DA) was used to detect the adulterated oregano samples, obtaining a 100% validation success rate, and partial least squares (PLS) analysis was used to quantify the percentage of adulterant. Finally, the proposed methodology was applied to 15 commercial oregano samples, resulting in two of them being classified as adulterated with 31 and 43% of olive leaves, respectively.

## 1. Introduction

Oregano is currently the best-selling herb due to its applications in the kitchen, where it is widely used as a condiment thanks to its great aromatic and flavoring qualities. The use of this herb is not only limited to culinary aspects but also extends to the cosmetic and pharmaceutical industries. It should be noted that oregano is an herb, not a spice; in fact, it is known as the “prince of the herbs.” Commercially, vegetable species with considerable amounts of carvacrol and sometimes thymol are called oregano [1,2].

To regulate the sale of this herb, two standards exist. While the ISO/FDIS 7925 [3] considers as true oregano the dried leaves and floral parts of all species of the *Origanum* genus (except *Origanum majorana* L.), the European Pharmacopeia [4] is more restrictive and only allows Mediterranean oregano (meaning Greek and Turkish oregano, *Origanum vulgare* L. ssp. *hirtum* and *Origanum onites* L., respectively) to be marketed as such. These standards are not always followed, and oregano is often adulterated for economic reasons (Economically Motivated Adulteration, EMA) [5]. This type of fraud usually consists of adding other species with lower commercial value, which are unspecified on the label, to the original product with the objective of obtaining greater profits [6]. Foods that frequently suffer from EMA have two common characteristics: high demand and price and a complex supply chain. Herbs and spices perfectly meet these criteria [2]. The demand for these products has increased so much in recent years that the global condiments market was worth €14.5 billion in 2019. Moreover, the condiments supply chain is described as “extremely complex” by the ESA (European Spice Association) [7]. In the case of oregano, these two characteristics add to the problem of defining its identity because of the great heterogeneity of the *Origanum* genus, which makes this herb even more vulnerable to fraud [2].

In the literature, there are many examples of oregano adulteration. In a study carried out between 2001 and 2007, 59% of the oregano samples contained extraneous material with a weight percentage greater than 20%. Almost none of the samples met the specifications of the ESA and ASTA (American Spice Trade Association), which consider impurities of a maximum of 2% to be tolerable [8,9,10]. More recently, a European report in 2021 made by the *Joint Research Center* (JRC) concluded that oregano was the most adulterated herb. Moreover, 48% of the analyzed oregano samples had undeclared material that, in most cases, was identified as olive leaves (*Olea europaea* L.) [11]. 

Companies test more and more of their products to fight against food fraud in the field of herbs and spices [7]. However, the detection of this illegal practice is an analytical challenge because there are a large number of adulterants that have been or could be used [5]. For this purpose, different methods have been developed. Black et al. [2] designed a qualitative method consisting of a screening using Fourier transform infrared spectroscopy (FT-IR) for the detection of adulterants in oregano, followed by a confirmatory assay with liquid chromatography (LC) coupled to high-resolution mass spectrometry (HRMS). Moreover, 24% of the studied samples were adulterated, and two out of seventy-eight had no oregano present. Wielogorska et al. [12] proposed a similar approach to the previous one but semi-quantitative, using FT-IR and LC with tandem mass spectrometry (MS/MS). Almost 90% of the analyzed oregano samples had at least one adulterant, and their weight percentage averaged 50%. Exclusive markers were used for each adulterant studied, and in the case of the olive and myrtle leaves, oleuropein was utilized. Bononi et al. also employed this marker to detect *Olea europaea* L. in oregano samples using LC-MS/MS with electrospray ionization (ESI) [13]. The monitoring of sorbitol by gas chromatography coupled to mass spectrometry (GC-MS) has also allowed the detection of the illegal presence of olive leaves in oregano samples [14]. Moreover, the combination of LC-MS/MS and GC-MS/MS enabled the identification of fraudulent samples using pesticides as markers of *Olea europaea* L. [15]. In a recent work, Creydt et al. [16] identified blumeatin as a marker to identify some common oregano adulterants using ion mobility MS coupled to LC. Other techniques that have been employed with the same purpose are direct analysis in real-time and an atmospheric solid analysis probe coupled to (HR)MS [5,17,18], proton nuclear magnetic resonance spectroscopy [19,20], photoacoustic laser spectroscopy [21], DNA-based methods [8,20,22,23,24,25] and near-infrared spectroscopy (NIR) [26,27,28,29]. 

Ion mobility spectrometry (IMS) has proved its great potential in the fight against food fraud by analyzing volatile organic compounds (VOCs) [30,31], but it has never been used for oregano quality control. Its fast response, high sensitivity, low operation cost, and minimal sample treatment explain the efficiency of this technique in many fields such as the agri-food and clinical fields [32]. The use of IMS as a GC detector increases the selectivity due to the previous separation of VOCs in the chromatographic column [33]. With GC-IMS, organic compounds are separated according to GC retention time and IMS drift time. The large amount of data provided by GC-IMS demands the use of data processing. In this work, the potential of IMS to evaluate the adulteration of oregano samples was explored for the first time. A non-targeted approach was applied to oregano VOC profiles obtained with headspace–gas chromatography coupled to ion mobility spectrometry (HS-GC-IMS) using chemometric tools such as orthogonal partial least squares-discriminant analysis (OPLS-DA) and partial least squares (PLS) to detect oregano samples adulterated with olive leaves and quantify the percentage of this adulterant, respectively. In addition, a targeted analysis was carried out, and some oregano and olive leaf volatiles were identified and quantified.

## 2. Materials and Methods

### 2.1. Reagents

For the study of VOC profiles of oregano and olive leaves, 57 compounds supplied by Sigma Aldrich (St. Louis, MO, USA) were used: twelve alcohols (ethanol, 2-methyl-1-butanol, 3-methyl-1-butanol, 1-pentanol, 1-penten-3-ol, cis-2-penten-1-ol, 1-hexanol, cis-2-hexen-1-ol, trans-2-hexen-1-ol, 2-heptanol, 2-octanol, 1-octen-3-ol), eleven ketones (2-butanone, 2-pentanone, 4-methyl-2-pentanone, 1-penten-3-one, 2-hexanone, 2-heptanone, 6-methyl-5-hepten-2-one, 2-octanone, 1-octen-3-one, 2-nonanone, 4-methylacetophenone), fourteen aldehydes (trans-2-pentenal, valeraldehyde, hexanal, heptanal, trans-2-heptenal, trans,trans-2,4-heptanodienal, octanal, trans-2-octenal, nonanal, trans-2-nonenal, decanal, trans-2-decenal, benzaldehyde, furfural), three aromatic hydrocarbons (2-pentylfuran, ethyl benzene, styrene), nine esters (ethyl acetate, pentyl acetate, hexyl acetate, ethyl butyrate, propyl butyrate, ethyl isovalerate, ethyl hexanoate, ethyl benzoate, oleuropein), and eight terpenes (carvacrol, thymol, linalool, limonene, γ-terpinene, terpinolene, p-cymene, sabinene). Many of these VOCs have previously been detected in olive leaves or oregano [34,35,36,37]. For each compound, a stock solution at 1000 µg g^−1^ was prepared in refined oil supplied by Sovena España S.A. (Brenes, Sevilla, Spain). These analytical standards were stored at 4 °C.

In order to verify the proper functioning of the HS-GC-IMS equipment, a standard solution at 0.3 µg g^−1^ of 2-butanone, 2-pentanone, 2-hexanone, 2-heptanone, and 2-octanone prepared in ultrapure water was injected before each sample sequence. Ultrapure water (18.2 MΩ cm^−1^) was obtained with a Milli-Q Plus system (Millipore, Bedford, MA, USA). The carrier and drift gas used in GC and IMS, respectively, was nitrogen, with a purity of 99.99% provided by Air Liquide (Madrid, Spain).

### 2.2. Oregano Samples

Ecological Mediterranean oregano employed was grown in Abanilla (Murcia, Spain). Olive leaves (*Olea europaea* L.) of two types, manzanilla and cornicabra, also harvested in Murcia (Spain), were used. Ecological oregano adulterated with different percentages of olive leaves (10, 20, 30, 40, and 50%) was analyzed with HS-GC-IMS. In addition, 15 commercial oregano samples (one of them purchased in bulk) obtained from local stores in Murcia were analyzed to implement the developed methodology.

### 2.3. Instrumentation and Software

The HS-GC-IMS equipment used was an Agilent Technologies 6890N gas chromatograph (Waldbronn, Germany) coupled to an IMS spectrometer from G.A.S (Dortmund, Germany). GC was equipped with a 2.5 mL syringe (Gerstel GmbH, Mühlheim, Germany) for headspace sampling and IMS with a tritium ionization source and a 98 mm length drift tube. Two capillary chromatographic columns from Agilent were tested: one non-polar HP-5MS-UI (5% diphenyl and 95% dimethylpolysiloxan) with 30 m length, 0.25 mm inner diameter, and 0.25 μm of film thickness; and other polar DB-WAX (100% polyethylene glycol) with the same dimensions as the previous one.

GC-IMS data were acquired with LAV version 2.1.1 (G.A.S, Dortmund, Germany). The statistical tools employed for data processing were SIMCA software version 14.1 (Umetrics, Malmö, Suecia), Statgraphics Centurion XV (StatPoint Technologies Inc., Warrenton, VA, USA), QtiPlot software trial version (Iondev Srl, Bucuresti, Rumania) and The Unscrambler X versión 10.4 (CAMO Software, Oslo, Norway).

### 2.4. HS-GC-IMS Method

A ground sample of 250 mg was placed into an 18 mL vial and incubated at 70 °C for 10 min at 750 rpm. Using *splitless* mode, the syringe at 80 °C injected 750 μL of headspace into the injector at 100 °C. Nitrogen with a constant flow rate of 1 mL min^−1^ was used as the carrier gas. The oven program started with a temperature of 50 °C maintained for 4 min, followed by an increase of 10 °C min^−1^ up to 130 °C, and, finally, this temperature was held for 8 min. The total GC run took 20 min, but the analysis, including incubation, required 30 min. After GC separation, analytes reached the IMS module in positive mode. The drift tube operated at 90 °C with a constant electric field of 500 V cm^−1^. Nitrogen gas drift constantly flowed at 150 mL min^−1^. Other IMS parameters were an average of 32 scans, a grid pulse width of 150 μs, a repetition rate of 30 ms, and drift and blocking voltages of 241 and 50 V, respectively.

### 2.5. Chemometric Data Processing

The HS-GC-IMS analysis results in a topographic map, a 3D spectrum in which the three variables are drift time in milliseconds, retention time in seconds, and intensity in volts. Firstly, all spectra of each oregano sample were aligned using one of them as reference. By means of a visual study, all the signals (a total of 449) that appeared in the topographic maps were manually selected. The intensity of all these markers was used as analytical signal and was obtained using LAV. Therefore, two datasets were created with the measured samples, and all markers selected in spectra resulted in two matrices with the following dimensions: 90 (samples) × 449 (markers) for OPLS-DA model and 60 (samples) × 449 (markers). Each dataset was randomly divided into two subsets: the calibration set, composed of 80% of the samples; and the validation set, composed of the remaining 20% [38].

**Figure 1:**
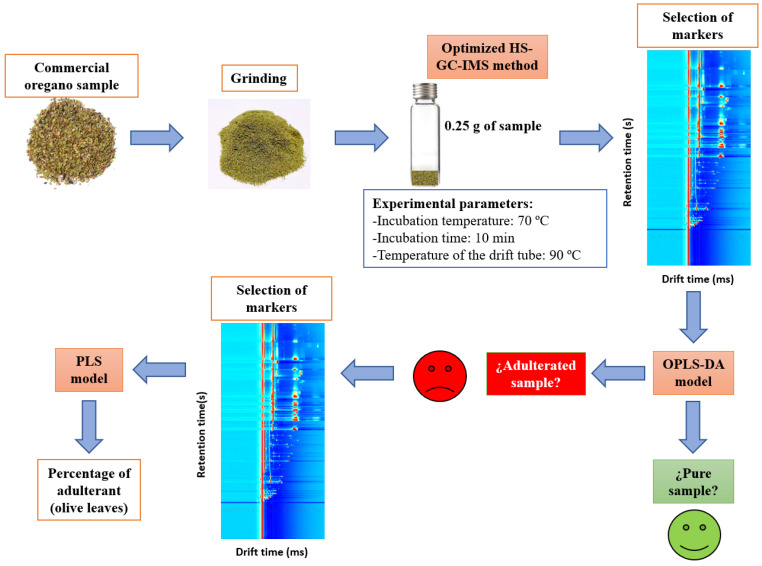
Summary of the proposed methodology.

Two different chemometric models based on OPLS-DA and PLS were developed, and several scaling methods, such as unit variance (UV) scaling and Pareto (Par) scaling, were tested. OPLS-DA was proposed to differentiate adulterated from unadulterated samples, while the PLS model was used to quantify the percentage of adulterant in samples classified as adulterated by OPLS-DA. All of the proposed methodology is summarized in Figure 1.

The prediction ability and the fit quality of OPLS-DA models were assessed with Q2 and R2, respectively. R2 is split into R2Y, which is the percentage of Y variation explained by the model, and R2X, which is the cumulative fraction of X variation. All these parameters take a maximum value of one, which means that the model is perfect [39]. A Q2 greater than 0.5 indicates that the prediction ability is acceptable, and if this parameter exceeds 0.9, the model is considered excellent [40]. The classification success rate, that is, the fraction of samples that a model correctly classifies, was also evaluated for both calibration and validation sets.

Furthermore, a PLS regression model was built using six percentages of olive leaves (0, 10, 20, 30, 40, and 50%). The model was assessed using the following quality parameters: the root-mean-square error of calibration (RMSEC) or validation (RMSEP), which measures the dispersion of the residuals; coefficient of determination (R^2^) and correlation, which evaluates linearity; and the standard error of calibration (SEC) or validation (SEP) and bias of validation or calibration, which check the accuracy. R^2^ must be close to one, and the remaining variables must approach zero for the model to be acceptable. RMSE and R^2^ are very useful to determine the number of optimal factors. Their values must be minimum for error and maximum for R^2^ to avoid overfitting and the introduction of noise into the model [41]. The factors were chosen using the representation of the error versus the number of factors.

## 3. Results and Discussion

### 3.1. Optimization of the HS-GC-IMS Method

The optimization of the HS-GC-IMS method was carried out using ecological Mediterranean oregano with the objective of obtaining a VOC profile with the maximum number of signals, which were as intense as possible and separated from each other.

Two capillary chromatographic columns were tested: a polar DB-WAX (100% polyethylene glycol) column and a non-polar HP-5MS-UI (5% diphenyl and 95% dimethylpolysiloxane) column. The polar one showed lower selectivity than the non-polar one because the signals overlapped more, especially at retention times longer than 500 s and drift times between 7 and 10 ms (Supplementary Materials Figure S1). For this reason, the non-polar column was chosen for the next experiences.

Subsequently, the amount of oregano and the sample incubation time and temperature were optimized together using a central composite face-centered design 2^3^ + star with three spaced central points due to the potential relationships between these parameters. This multivariate study generated 17 experiments to be carried out with HS-GC-IMS where the variables were modified in the following ranges: incubation time between 1 and 15 min, incubation temperature between 60 and 90 °C, and sample amount between 0.1 and 0.5 g. After selecting all the signals, their intensities were calculated and added so that the response variable was the sum of all of them. Results fit perfectly with the response surface shown in Supplementary Materials Figure S2 because R^2^ was 0.98. The optimal values obtained were the highest studied because this design only considers the intensity of the markers. Using these values, the degree of overlap was considerable, and the equipment became very dirty, requiring four blanks between samples. Each parameter was, therefore, optimized separately in the same above-mentioned ranges. Higher values of the variables were not studied because the overlap of signals would be more pronounced, and the instrument would become dirtier.

**Figure 2:**
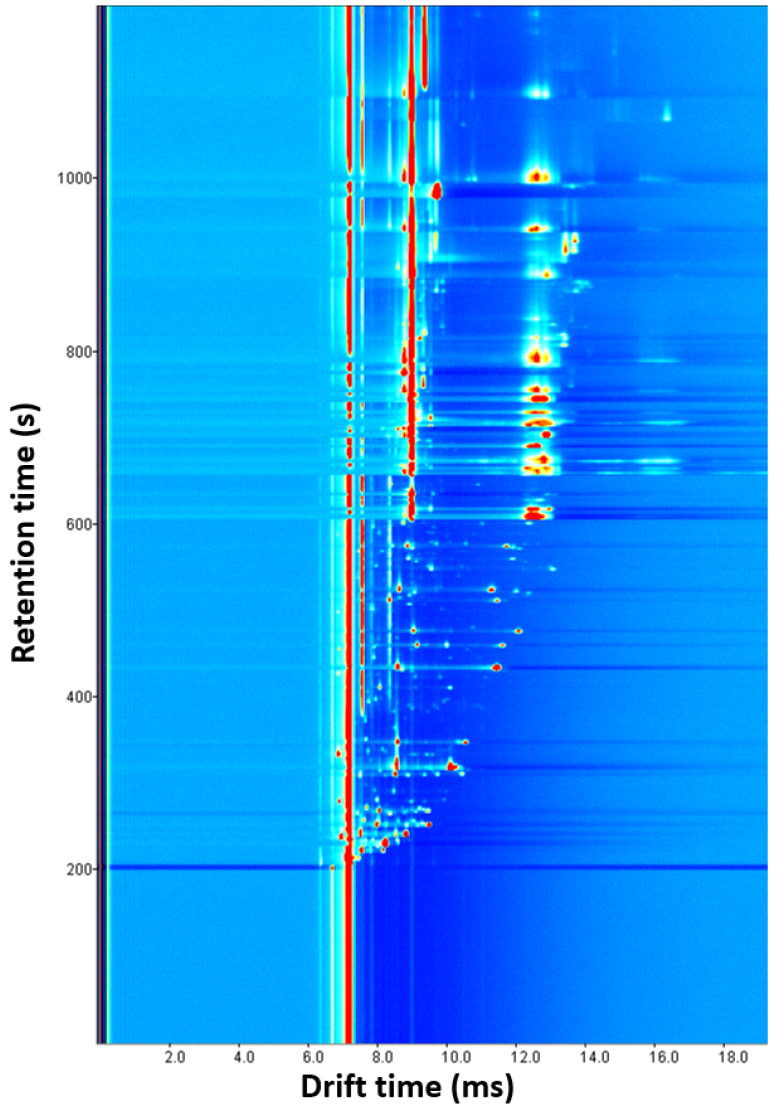
Ecological oregano spectrum obtained with the optimized HS-GC-IMS method.

Three experiments were conducted by changing the amount of oregano (0.1, 0.25 and 0.5 g). As the amount increased, the number and intensity of signals increased. Several overlaps were observed with 0.5 g, and some signals disappeared with 0.1 g, so 0.25 g was chosen as the optimal sample amount (Supplementary Materials Figure S3). With this parameter optimized, four experiments were carried out by modifying the incubation time (1, 5, 10, and 15 min). The release of VOCs was favored given a longer time. Since there were no significant differences between 10 and 15 min and short analysis times are preferred, 10 min was chosen as the optimal value (Supplementary Materials Figure S4). Next, the incubation temperature was optimized by performing four experiments (60, 70, 80, and 90 °C) in which the incubation time and the amount of sample were 10 min and 0.25 g, respectively (Supplementary Materials Figure S5). The signals became more intense after increasing temperature, but more blanks between samples were required. The higher intensity and the minimum number of blanks, three, were achieved at 70 °C, the optimal value. Finally, with the previously optimized parameters, the drift tube temperature was studied with three experiences (70, 80 and 90 °C). As shown in Supplementary Materials Figure S6, more intense signals without overlapping were obtained at 90 °C, so this was selected as the optimal temperature. Higher values could not be tested because the drift tube supports a maximum temperature of 100 °C. Figure 2 shows the ecological oregano spectrum obtained with the developed method. Despite optimization, a lot of signals appeared overlapped at a drift time of 9 ms and from a 600 s retention time.

### 3.2. Identification of VOCs

After optimization, the identification of the greatest possible number of signals was carried out on topographic maps of ecological oregano and the two types of olive leaves. For this purpose, a total of 57 standard solutions of compounds specified in Section 2.1 were prepared at 1000 µg g^−1^ in refined oil since water could not be used as a solvent in the solutions because 15 of the 30 identified compounds are not water-soluble (Supplementary Materials Table S1). Compounds with a solubility lower than 0.1 g/100 mL were considered insoluble [42].

Dilutions at 10 µg g^−1^ were prepared and measured with the optimized HS-GC-IMS method. In the cases of carvacrol and thymol, solutions at 1000 µg g^−1^ were used because no signals were observed at 10 µg g^−1^. The quantity of solutions transferred to the vials was the same as the optimized amount of sample, which was 0.25 g. A total of 30 compounds were present in oregano and/or olive leaves. The identification was based on the retention and drift times of VOCs (Supplementary Materials Table S2). Ethanol and oleuropein produced exactly the same protonated monomer and proton-bound dimer signals, so they were ruled out due to a lack of selectivity. Despite this, the developed method allows one to separate, monitor, and identify 30 volatiles at the same time.

**Figure 3:**
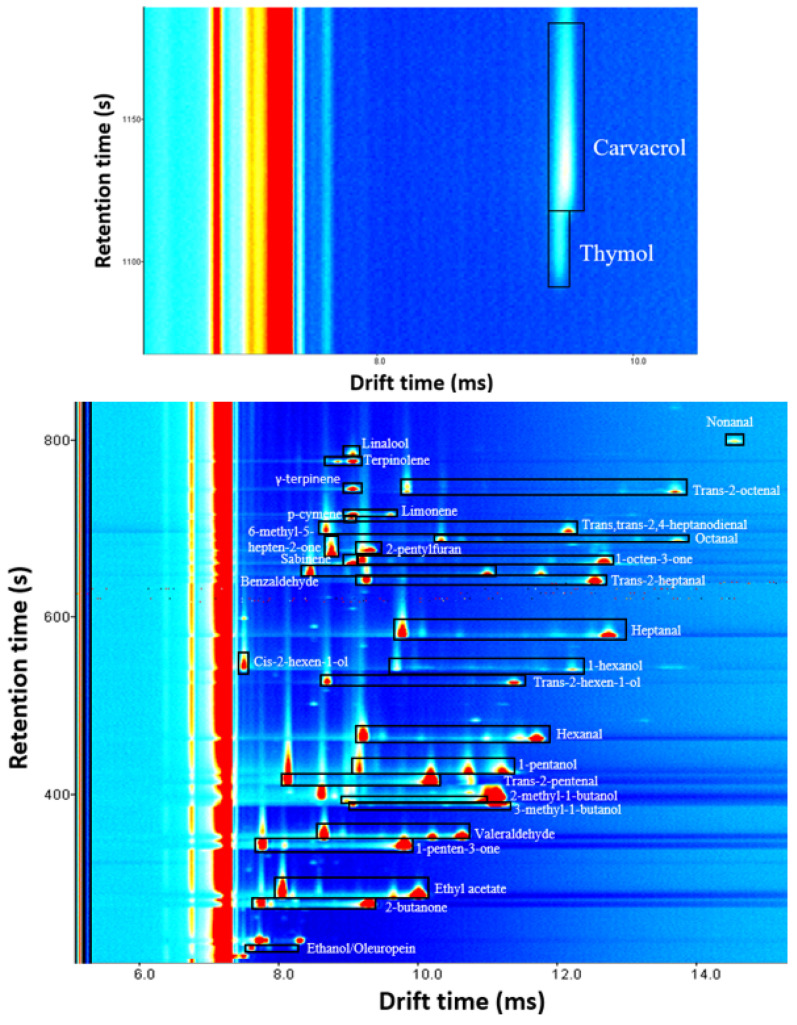
Signal distribution of compounds identified in oregano and olive leaves. This spectrum was the result of analyzing a mixed solution of all identified volatiles with a concentration of 10 µg g^−1^ in all cases except for carvacrol and thymol, which were measured at 1000 µg g^−1^. Protonated monomer and proton-bound dimer signals of each compound are inside a black rectangle. The unselected signals were produced by refined oil.

Figure 3 shows the signal distribution of identified compounds. A visual exploration of the spectra reveals that almost all VOCs present proton-bound dimer and protonated monomer at the used concentration, with the exception of nonanal, cis-2-hexen-1-ol, 6-methyl-5-hepten-2-one, 2-pentylfuran, p-cymene, γ-terpinene, linalool, thymol and carvacrol which have only protonated monomer.

### 3.3. Characterization of HS-GC-IMS Method

In order to validate the method, a precision study was carried out and calibration curves were built. Firstly, precision was assessed in terms of repeatability (or intraday precision) and intermediate (or interday) precision using the relative standard deviation (RSD) of the signal intensity. To evaluate the repeatability, a mixed standard of all identified compounds was analyzed five times at two different concentrations on the same day, while to evaluate intermediate precision, the mixture was analyzed three times at the same two levels of concentration on three different days. The levels of concentration were 1 and 5 µg g^−1^ for all compounds, except for nonanal (7.5 and 15 µg g^−1^), thymol (2500 and 5000 µg g^−1^) and carvacrol (5000 µg g^−1^). As can be observed in Supplementary Materials Table S3, good precision results were obtained. The intraday RSD varied between 0.5 and 11.6%, while the interday RSD ranged from 1.5 to 13.3%.

Calibration curves of the 30 identified compounds were then assessed. To construct them, mixed standards at different concentrations between 0.1 and 10 µg g^−1^ (except for carvacrol and thymol, which require concentrations higher than 1000 µg g^−1^ to produce a quantifiable signal) were analyzed with the optimized method. According to previous works [43], a logarithmic fit was performed using different analytical responses of the HS-GC-IMS analysis: protonated monomer intensity, proton-bound dimer intensity, and the sum of both. Coefficients of determination for these adjustments are shown in Supplementary Materials Table S4. To improve the quality of the regression, the above-mentioned analytical responses were fitted to Boltzmann’s equation, which is as follows:

$$y=A2+\frac{A1-A2}{1+e^{[\left(lnx-x0\right)/dx]}}$$

*A*2, *A*1, *dx*, and *x*0 are constants. Supplementary Materials Table S5 shows R^2^ of these adjustments, which are higher than those obtained with the logarithmic fit. This type of adjustment gave rise to very high coefficients in all cases. There were practically no differences between using the proton-bound dimer signal when it was present or using the sum of protonated monomer and proton-bound dimer signals. However, since the proton-bound dimer signal did not appear in all the identified compounds, it was decided to use the sum of both signals in all cases. When the proton-bound dimer signal was not present, the regression of the protonated monomer and the sum of the protonated monomer and proton-bound dimer signals coincided. The constants for these adjustments are given in Supplementary Materials Table S6.

The limits of detection (LOD) and limits of quantification (LOQ) were calculated as 3 and 10 signal-to-noise (S/N) ratios, respectively. These calculations were carried out using the protonated monomer signal because the proton-bound dimer signal disappears at low concentrations. As can be seen in Supplementary Materials Table S7, the LOD varies between 0.02 and 298 µg g^−1^, and the LOQ ranges between 0.08 and 994 µg g^−1^. These values correspond to 1-penten-3-one and thymol, respectively.

### 3.4. Quantification of Identified VOCs

Calibration curves were then applied for the quantification of volatiles in ecological oregano samples adulterated with known percentages of the two types of olive leaves. The studied percentages were 0, 10, 20, 30, 40, and 50%. For each of them, 10 measurements with the HS-GC-IMS method were made, half with cornicabra olive leaves and half with manzanilla olive leaves. At a drift time of 9 ms and from a 600 s retention time, many overlapping signals appeared (Figure 2). This fact did not allow the quantification of the following volatiles: sabinene, p-cymene, γ-terpinene, and linalool. In addition, the proton-bound dimer of terpinolene and the protonated monomer of limonene also overlapped in this region of the spectrum, but in these cases, the protonated monomer of terpinolene and the proton-bound dimer of limonene could be used for quantification. In the same way, proton-bound dimers of 2-methyl-1-butanol and 3-methyl-1-butanol overlap (Figure 3), so quantification must be carried out only using the protonated monomer signal of both. On the other hand, carvacrol and thymol produced signals that saturated the IMS spectrometer, so these compounds could not be quantified. In conclusion, 24 out of 30 identified VOCs were quantifiable.

Note that calibration curves were carried out in refined oil due to its solubility, as previously mentioned, but the oregano samples were analyzed directly. Therefore, in order to correct possible variations in the signals due to the difference in the matrices, experiences were made by adding 0.25 g of refined oil to 0.25 g of ecological oregano to evaluate the differences in the signals. When oil was added, the intensity of signals was halved; therefore, a correction factor of 0.5 was applied to the signals of oregano samples to be able to use the calibration curves prepared with refined oil as a solvent.

Average concentrations of compounds with their standard deviations in ecological oregano samples with different percentages of olive leaves are shown in Table 1. It should be noted that the standard deviation obtained in adulterated samples was higher than in pure samples due to the use of two types of olive leaves whose VOC content is different. Nonanal, trans-2-octenal and 2-pentylfuran were not detected in the samples. These compounds were identified in olive leaves, but when the percentage of this adulterant was halved, the signals disappeared. On the other hand, the signal intensities of terpinolene and limonene were too intense to be quantified and were outside the range of intensities in which the calibration has been performed, so the concentration of these compounds was higher than 10 µg g^−1^ (the highest concentration point of the calibration). Supplementary Materials Figure S7 shows how the concentration of compounds changed with the percentage of adulterants. By increasing the content of olive leaves, the concentration of trans-2-hexen-1-ol decreased, and other compounds, such as cis-2-hexen-1-ol and 6-methyl-5-hepten-2-one, kept their concentrations constant.

With the purpose of establishing specific markers that allow adulterated samples to be identified, a one-way analysis of variance (ANOVA) was performed. Concentrations of compounds in pure and adulterated samples were compared. Significant differences between these two categories were found in the following cases: 2-butanone (*p*-value = 0.0000), trans-2-hexen-1-ol (*p*-value = 0.0000), benzaldehyde (*p*-value = 0.0093), hexanal (*p*-value = 0.0482), limonene (*p*-value = 0.0088), trans-2-pentenal (*p*-value = 0.0155), and valeraldehyde (*p*-value = 0.0318). Although some important differences have been observed due to the high variability between samples, no rules could be created to distinguish the two categories. The solution was the development of chemometric models.

### 3.5. Chemometric Models

#### 3.5.1. OPLS-DA for the Detection of Samples Adulterated with Olive Leaves

**Figure 4:**
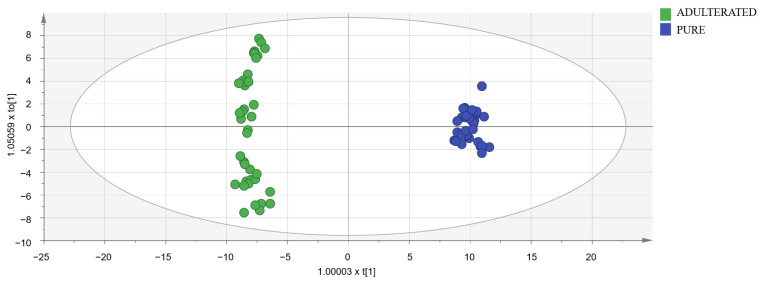
Score-plot of the selected OPLS-DA model.

To detect oregano samples adulterated with olive leaves, OPLS-DA models were designed using a data matrix with the following dimensions: 90 samples (50 adulterated samples and 40 pure samples) × 449 markers selected in spectra after a visual exploration. Adulterated samples set was formed by 10 measurements (five with cornicabra olive leaves and five with manzanilla olive leaves) of each of the following percentages: 10, 20, 30, 40, and 50%. Models were constructed using the calibration set (40 adulterated samples and 32 pure samples). UV and Par scaling were tested and obtained almost identical results. Then, UV scaling was chosen because it is the most common and well-known scaling method for metabolomic data. Subsequently, models were validated with the validation set (ten adulterated samples and eight pure samples). The importance of markers was studied by means of VIP (variable importance for the projection) [44]. Markers with a high value of this parameter are really useful for distinguishing between pure and adulterated samples. The best results in terms of Q2, R2X, and R2Y were obtained with the 114 markers with the highest VIP (Supplementary Materials Table S8). The need for logarithmic transformation was also evaluated. In the normal probability plot, residuals were best fitted to a straight line using the logarithmic transformation, so data were not normally distributed, and the transformation was necessary. In conclusion, the best model (shown in Figure 4) utilized 114 markers and the logarithmic transformation. Its classification success rate was 100% in both the calibration and validation sets. As it had a Q2 higher than 0.9, the selected model was excellent [40].

In the loading plot of the selected OPLS-DA model (Supplementary Materials Figure S8), it can be seen that markers corresponding to compounds like 6-methyl-5-hepten-2-one, 1-pentanol, trans,trans-2,4-heptanodienal, 3-methyl-1-butanol, and limonene are more intense in adulterated samples, while markers of 2-butanone, ethyl acetate, and trans-2-pentenal are more intense in pure samples. These differences agree with the result shown in Table 1. Unfortunately, none of the most important markers to differentiate the two classes have been identified.

Another way to evaluate the developed model is to use the permutation plot (Supplementary Materials Figure S9). This validation consists of comparing the R2 and Q2 values of the original model with the values of other models designed with the same data but with random permutations of Y observations. In Supplementary Materials Figure S9, 50 permutations were carried out. The vertical axis represents the R2 and Q2 values of the original model (located further to the right) and the models obtained with the permutations. The horizontal axis represents the correlation between permuted and original Y (corr(Y,Y_perm_)) [45]. A model is acceptable when all the values of R2 and Q2 are below the original values, and the regression line of Q2 points intersects the vertical axis below zero. The selected model fulfills all these criteria, so it is valid.

#### 3.5.2. PLS for Quantification of the Olive Leaves Content in Oregano Samples

After detecting adulterated oregano samples using the OPLS-DA model, it could be of interest to know the magnitude of the adulteration with olive leaves. With this purpose, a PLS model was developed using a data matrix with the following dimensions: 60 samples × 449 markers. Specifically, 10 oregano samples with each of the following six percentages of olive leaves were used: 0, 10, 20, 30, 40, and 50%. This sample set was split into two groups: the calibration set comprising 48 samples (80%) and the validation set with 12 samples (20%). Supplementary Materials Figure S10 shows the linear regression obtained with the calibration and validation set. The optimal number of factors (chosen according to RMSE and R^2^, Section 2.5) was 25, including 99% of the accumulated variance. Supplementary Materials Table S9 shows the very good quality parameters of this model, among which the R^2^ of calibration of 0.9999998 stands out. The results of the validation are summarized in Supplementary Materials Table S10. As can be seen, percentages predicted by the PLS model matched the real percentages of olive leaves with a standard deviation ranging between 0.9 and 2%.

### 3.6. Analysis of Commercial Oregano Samples and Comparison with Other Reported Methodologies

The proposed methodology summarized in Figure 1 was applied to study the authenticity of the 15 commercial oregano samples specified in Section 2.2. Firstly, the concentration of the 24 quantifiable VOCs was determined (Supplementary Materials Table S11). The high content of some compounds, such as hexanal and trans-2-hexen-1-ol, should be noted.

Subsequently, the OPLS-DA model was applied. It was concluded that 13 of the 15 samples were pure, and the remaining two were classified as adulterated. Following the scheme in Figure 1, the PLS model was applied to the two adulterated samples. The percentages of olive leaves predicted by this chemometric tool were 31 ± 12% and 43 ± 11%. These percentages are not higher than other amounts of adulterant found in the literature. Black et al. [2] reported that 2 out of 78 analyzed samples had no oregano present, and Wielogorska et al. [12] found that the adulterant percentage averaged 50% in fraudulent oregano samples. In the first work, a long extraction step for the LC analyses was required, which lengthened the methodology. The second work shortened the pretreatment proposed by Black et al., but in both methods, organic solvents were used in extraction and chromatography. The methodology proposed in this study employs a minimal sample pretreatment and no solvent, so it can be included in the green analytical chemistry.

On the other hand, NIR-based methods use no sample pretreatment, but they have limited usefulness in solving the authentication problems of oregano. The NIR-based methodology proposed by Rodionova et al. [27] is “only partially appropriate” to discriminate between adulterated and nonadulterated oregano samples, and the proposed one by McVey et al. [29] correctly predicts 100% of adulterant samples and 90% of authentic samples. With the HS-GC-IMS method of this paper, 100% of genuine or adulterated oregano samples are successfully classified, and, in contrast to the last cited article, the percentage of adulterants can be quantified. In the same way, reported ambient mass spectrometry methods have not been able to determine the amount of adulteration [5,17,18]. DNA-based methods are more time-consuming, and in some cases, if the presence of the adulterant is detected, it can be due to an “(insignificant) trace contamination” [25]. Parveen et al. [46] explained other limitations of DNA-based techniques in the field of herbs authentication.

## 4. Conclusions

In this work, the potential of HS-GC-IMS analysis followed by chemometric treatment for oregano quality control has been demonstrated. Using the developed OPLS-DA model, oregano samples adulterated with olive leaves can be detected. Subsequently, the PLS model could be applied to quantify the percentage of olive leaves. These two chemometric models have excellent quality parameters, among which the validation success rate (100%) for the OPLS-DA and R^2^ of calibration (0.9999998) for PLS stand out. This proposed methodology was used in the study of the authenticity of fifteen commercial oregano samples, resulting in two of them being classified as adulterated with 31 and 43% of olive leaves, respectively. Therefore, oregano quality is still a problem in the market where high levels of adulteration ensure high economic profits for fraudsters.

On the other hand, thirty compounds were identified in the VOC profiles of ecological Mediterranean oregano and the two types of olive leaves, which are cornicabra and manzanilla. However, only 24 compounds could be quantified using calibration curves based on Boltzmann’s equation.

The excellent results obtained together with the low operation cost, the minimal sample treatment, and the short analysis time (30 min) make the proposed methodology a very useful and robust tool for use in the agri-food industry. This work helps to solve the problem of oregano adulteration with olive leaves. Unfortunately, the number of adulterants that have been used or can be used is large, which complicates the development of methods to detect adulterated oregano samples. More research about the detection of adulterants in oregano samples is required, and, in those investigations, IMS coupled to GC could play an important role due to its great advantages in the VOC analysis, as demonstrated by this work.

## Figures and Tables

**Table 1:** Concentrations of volatile compounds in ecological oregano samples adulterated with different percentages of olive leaves (µg g^−1^).

Compound	Percentages of Olive Leaves					
	0%	10%	20%	30%	40%	50%
2-butanone	0.138 ± 0.005	0.11 ± 0.02	NQ	NQ	NQ	NQ
Ethyl acetate	NQ	NQ	NQ	NQ	NQ	NQ
1-penten-3-one	NQ	NQ	NQ	NQ	NQ	NQ
Valeraldehyde	0.55 ± 0.02	0.9 ± 0.3	0.9 ± 0.3	0.7 ± 0.2	0.7 ± 0.2	0.6 ± 0.1
3-methyl-1-butanol	ND	NQ	NQ	NQ	NQ	NQ
2-methyl-1-butanol	ND	ND	NQ	NQ	NQ	NQ
Trans-2-pentenal	0.503 ± 0.011	0.48 ± 0.03	0.43 ± 0.09	0.4 ± 0.1	0.4 ± 0.1	0.43 ± 0.08
1-pentanol	ND	NQ	NQ	NQ	NQ	NQ
Hexanal	1.19 ± 0.08	2.3 ± 0.7	1.8 ± 1.2	1.7 ± 1.2	1.6 ± 1.1	1.8 ± 0.9
Trans-2-hexen-1-ol	8.9 ± 0.5	7.6 ± 0.6	6.4 ± 1.7	6 ± 2	5.1 ± 1.9	4.7 ± 1.2
1-hexanol	ND	ND	ND	ND	NQ	NQ
Cis-2-hexen-1-ol	0.529 ± 0.015	0.53 ± 0.05	0.51 ± 0.04	0.51 ± 0.07	0.55 ± 0.10	0.54 ± 0.10
Heptanal	0.107 ± 0.005	0.114 ± 0.006	NQ	NQ	NQ	0.12 ± 0.03
Trans-2-heptenal	ND	NQ	NQ	NQ	NQ	NQ
Benzaldehyde	0.741 ± 0.016	0.825 ± 0.019	0.86 ± 0.09	0.87 ± 0.15	0.9 ± 0.2	0.9 ± 0.2
1-octen-3-one	2.16 ± 0.06	2.3 ± 0.2	2.14 ± 0.18	2.1 ± 0.3	2.0 ± 0.3	2.0 ± 0.2
6-methyl-5-hepten-2-one	0.99 ± 0.06	0.93 ± 0.12	0.97 ± 0.15	1.0 ± 0.3	1.0 ± 0.4	0.9 ± 0.3
2-pentylfuran	ND	ND	ND	ND	ND	ND
Octanal	ND	ND	NQ	NQ	NQ	NQ
Trans,trans-2,4-heptanodienal	NQ	NQ	NQ	NQ	NQ	NQ
Limonene	>10	>10	>10	>10	>10	>10
Trans-2-octenal	ND	ND	ND	ND	ND	ND
Terpinolene	>10	>10	>10	>10	>10	>10
Nonanal	ND	ND	ND	ND	ND	ND

ND: not detected, NQ: detected, but not quantified.

## Data Availability

Data is contained within the article or Supplementary Material, further inquiries can be directed to the corresponding author.

## Supplementary Materials

The following supporting information can be downloaded at https://www.mdpi.com/article/10.3390/foods13040516/s1, Figure S1: HS-GC-IMS spectra with the polar and non-polar column, Figure S2: Estimated response surface obtained using a central composite face-centered design 2^3^ + star with three spaced central points for the optimization of three variables together: amount of sample and incubation time and temperature, Figure S3: Enlargement of HS-GC-IMS spectra obtained for different amounts of samples at retention times between 200 and 400 s, Figure S4: Enlargement of HS-GC-IMS spectra obtained for different incubation times at retention times between 200 and 400 s, Figure S5: Enlargement of HS-GC-IMS spectra obtained for different incubation temperatures at retention times between 200 and 400 s, Figure S6: Enlargement of HS-GC-IMS spectra obtained for different temperatures of the drift tube at retention times between 200 and 550 s, Figure S7: Variation of concentration of VOCs with the percentage of olive leaves, Figure S8: Loading-plot of the selected OPLS-DA model with labels in the markers corresponding to identified compounds, Figure S9: Permutation plot for the selected OPLS-DA model. A total of 50 permutations were carried out, Figure S10, PLS regression both with calibration set (blue) and with validation set (red); Table S1: Water solubility of identified compounds, Table S2: VOCs identified in ecological Mediterranean oregano and olive leaves (manzanilla or cornicabra), Table S3: Precision study with identified compounds (RSD, %), Table S4: R^2^ of calibration curves obtained with logarithmic regression adjustment, Table S5: R^2^ of calibration curves obtained with the adjustment to Boltzmann’s equation, Table S6: Constants of the calibration curves obtaining with the adjustment of the sum of the intensities of protonated monomer and proton-bound dimer to Boltzmann’s equation, Table S7: LOD and LOQ of the developed method, Table S8: Information about OPLS-DA models, Table S9: Information about PLS model, Table S10: Validation of PLS model, Table S11: Quantification of VOCs in commercial oregano samples (μg g^−1^).
