# Supplementary material for: Detection of Adulterated Oregano Samples Using Untargeted Headspace–Gas Chromatography–Ion Mobility Spectrometry Analysis
Source: Foods. 2024 Feb 7;13(4):516. doi: 10.3390/foods13040516 (PMC10888469; doi:10.3390/foods13040516)
Supplement: Supplementary file 1 [file foods-13-00516-s001.zip › foods-2842235-supplementary.pdf]

foods

Supplementary Material

Detection of adulterated oregano samples using untargeted Headspace - Gas Chromatography - Ion Mobility Spectrometry analysis

Blas Rocamora-Rivera, Natalia Arroyo-Manzanares*, Pilar Viñas

Department of Analytical Chemistry, Faculty of Chemistry, University of Murcia, 30100 Murcia, Spain; blas.rocamorar@um.es (B.R.-R.); pilarvi@um.es (P.V.)
* Correspondence: natalia.arroyo@um.es (N.A.-M.)

Received: date; Accepted: date; Published: date

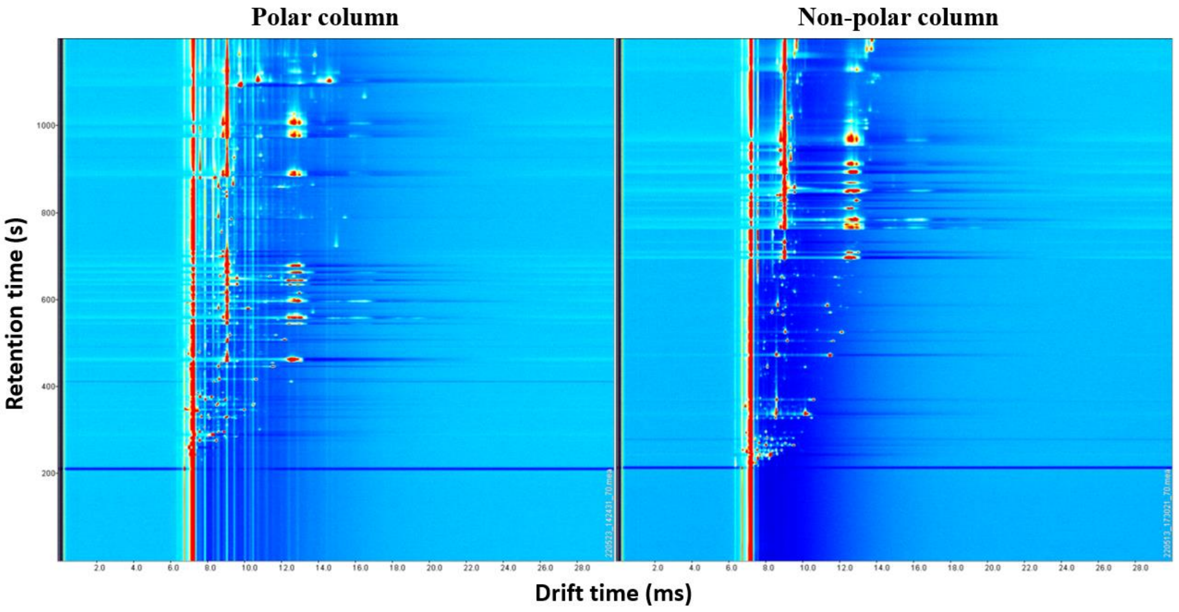

Supplemental Figure S1. HS-GC-IMS spectra with the polar and non-polar column.

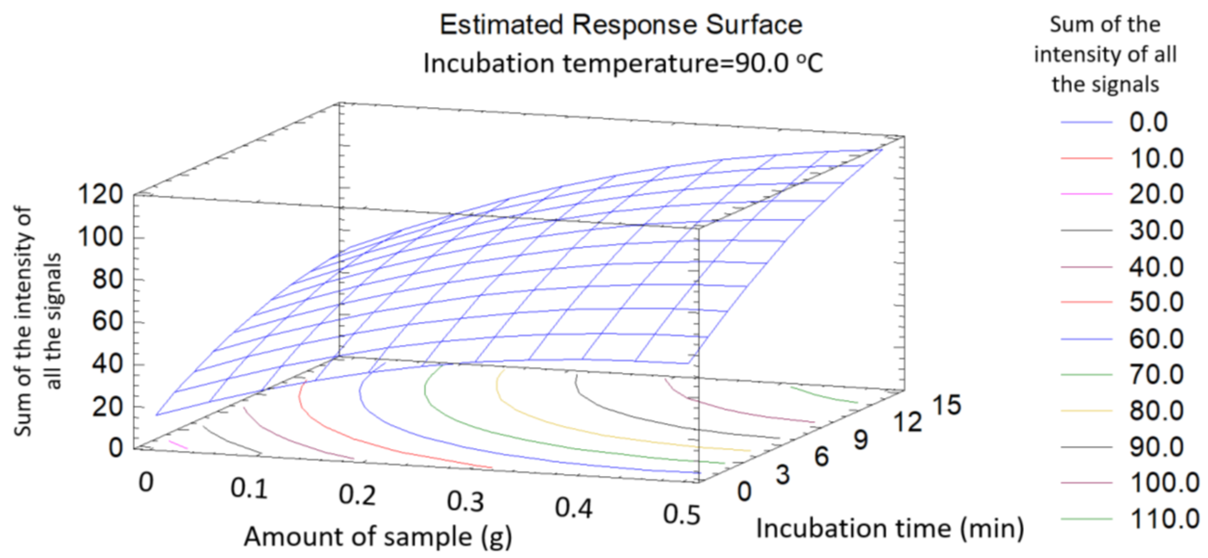

Supplemental Figure S2. Estimated response surface obtained using a central composite face-centered design 2^3 + star with three spaced central points for the together optimization of three variables: amount of sample and incubation time and temperature.

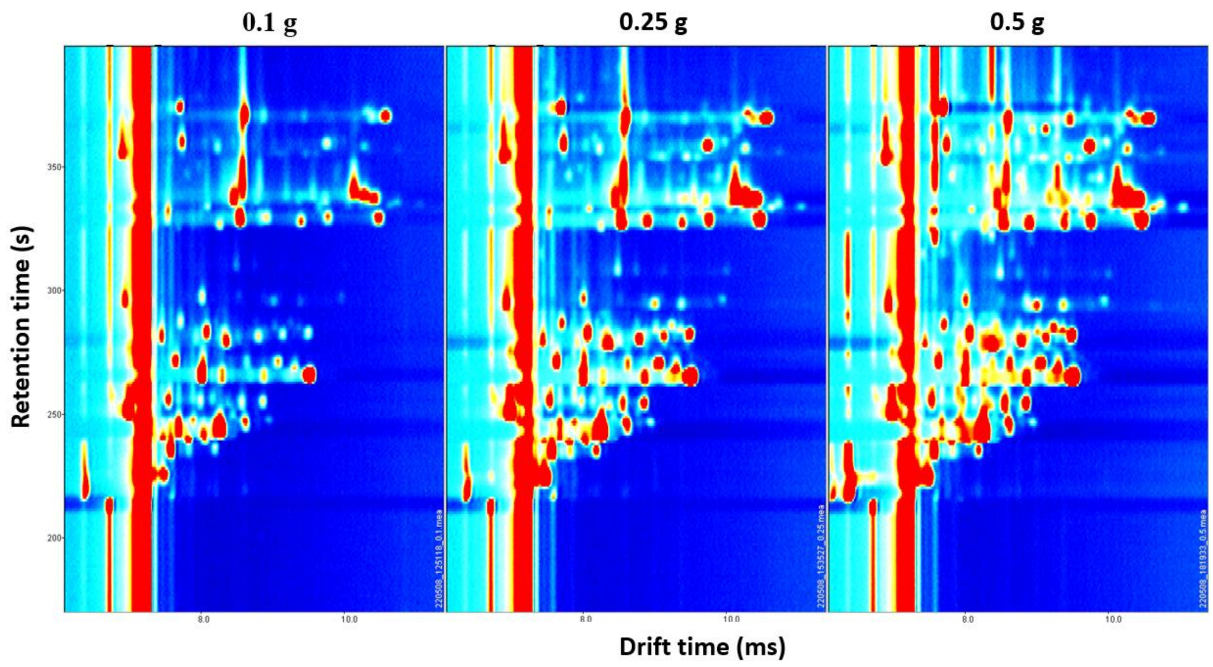

Supplemental Figure S3. Enlargement of HS-GC-IMS spectra obtained for different amounts of samples at retention times between 200 and 400 s.

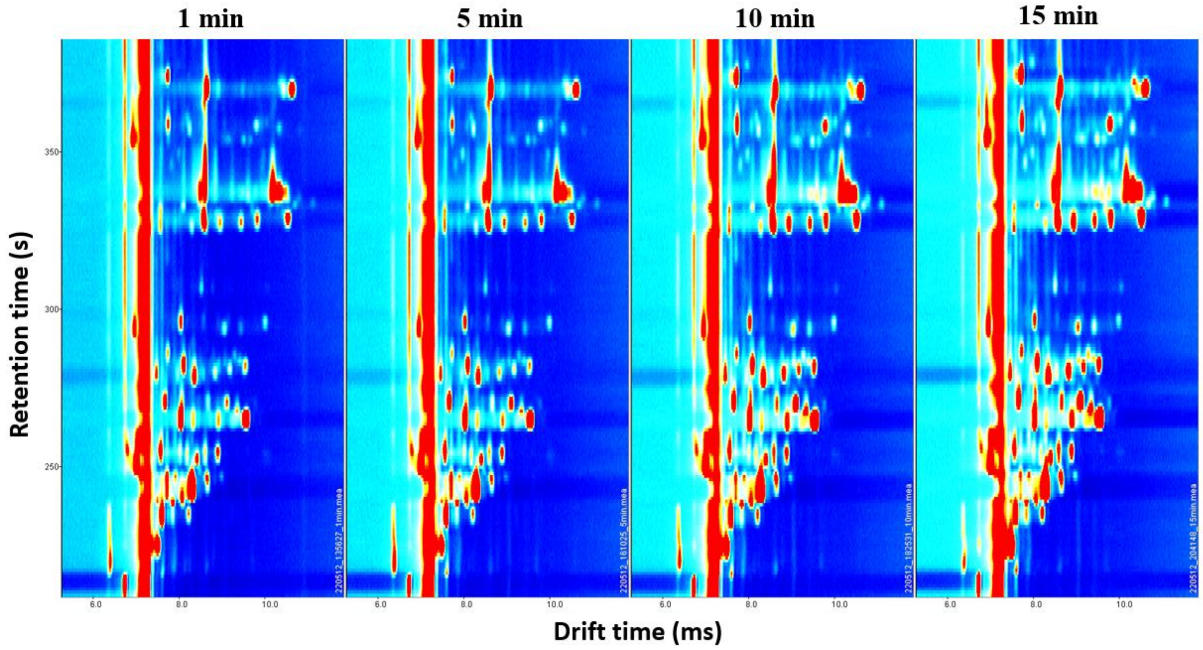

Supplemental Figure S4. Enlargement of HS-GC-IMS spectra obtained for different incubation times at retention times between 200 and 400 s.

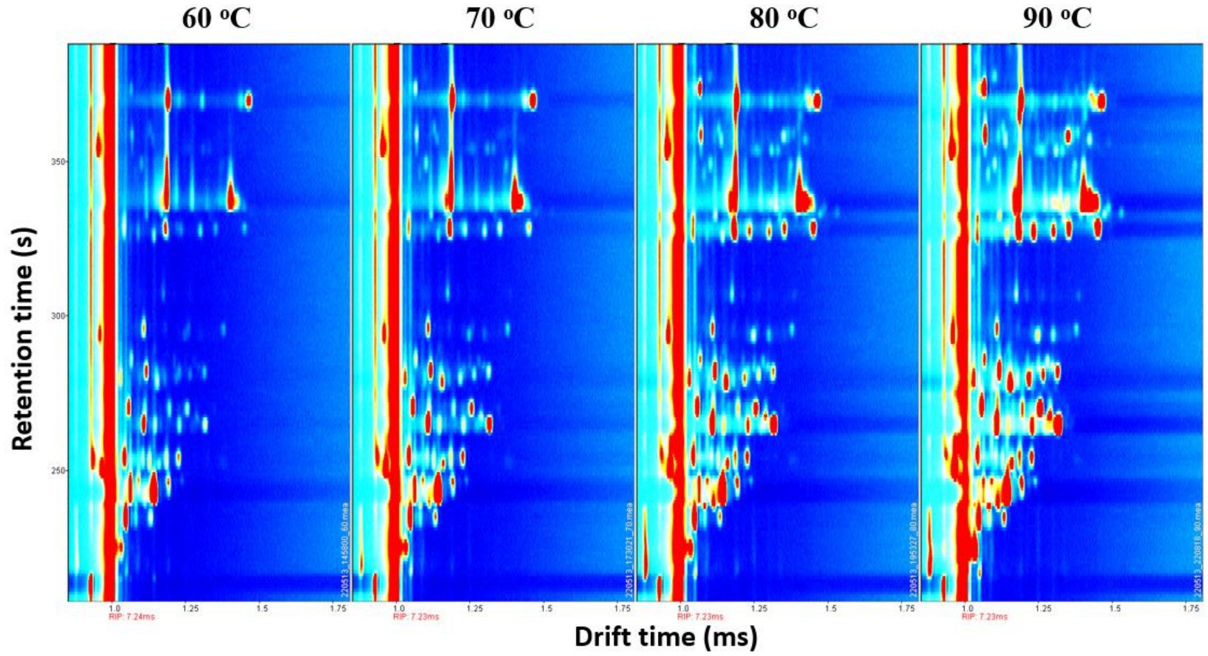

Supplemental Figure S5. Enlargement of HS-GC-IMS spectra obtained for different incubation temperatures at retention times between 200 and 400 s.

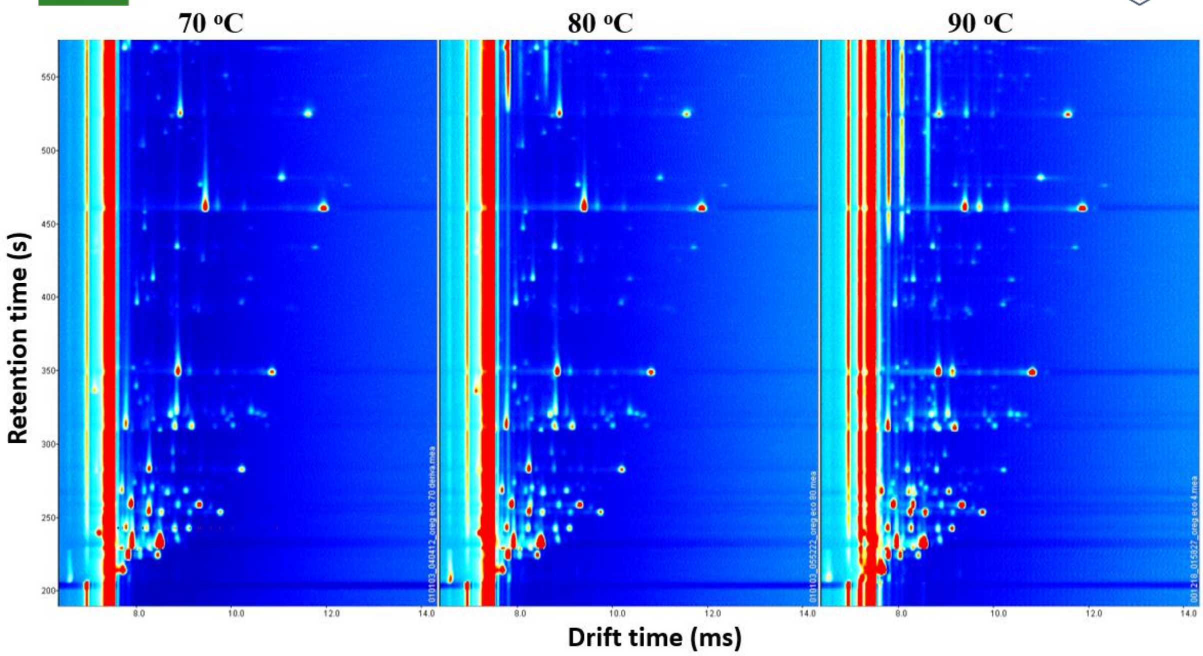

Supplemental Figure S6. Enlargement of HS-GC-IMS spectra obtained for different temperatures of the drift tube at retention times between 200 and 550 s.

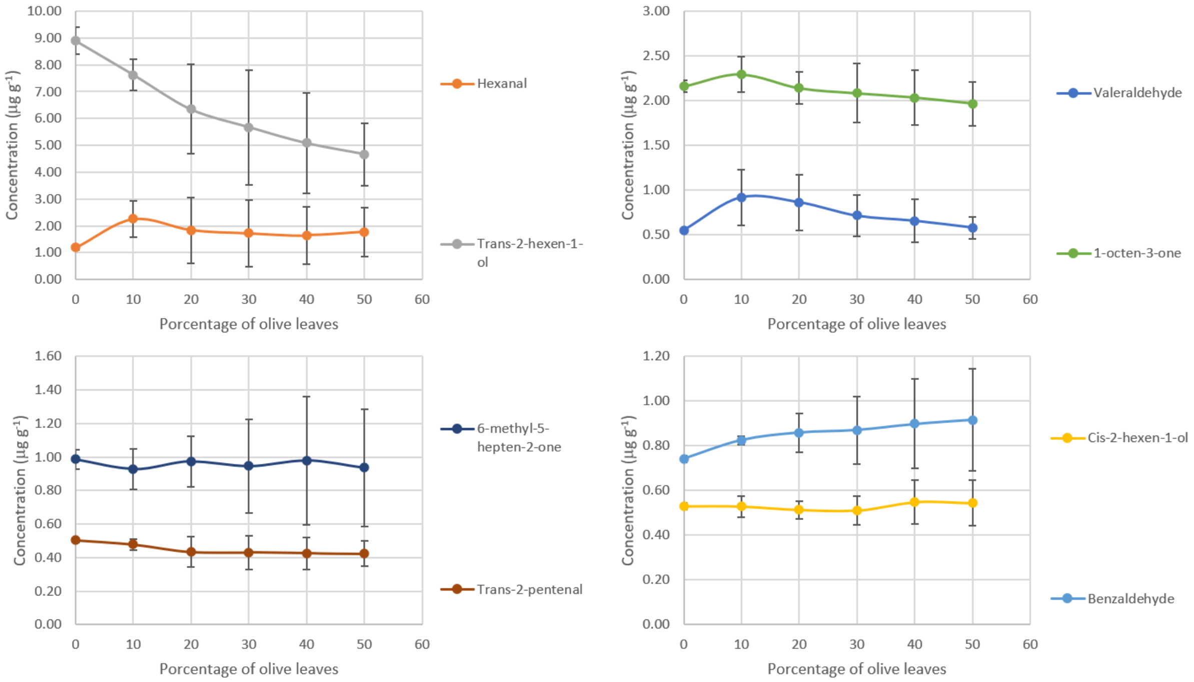

Supplemental Figure S7. Variation of concentration of VOCs with the percentage of olive leaves.

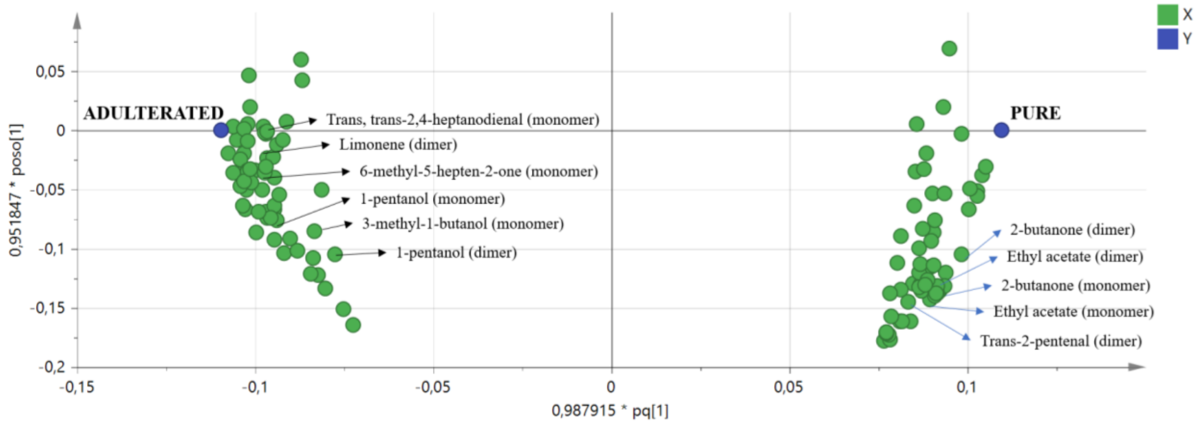

Supplemental Figure S8. Loading-plot of the selected OPLS-DA model with labels in the markers corresponding to identified compounds.

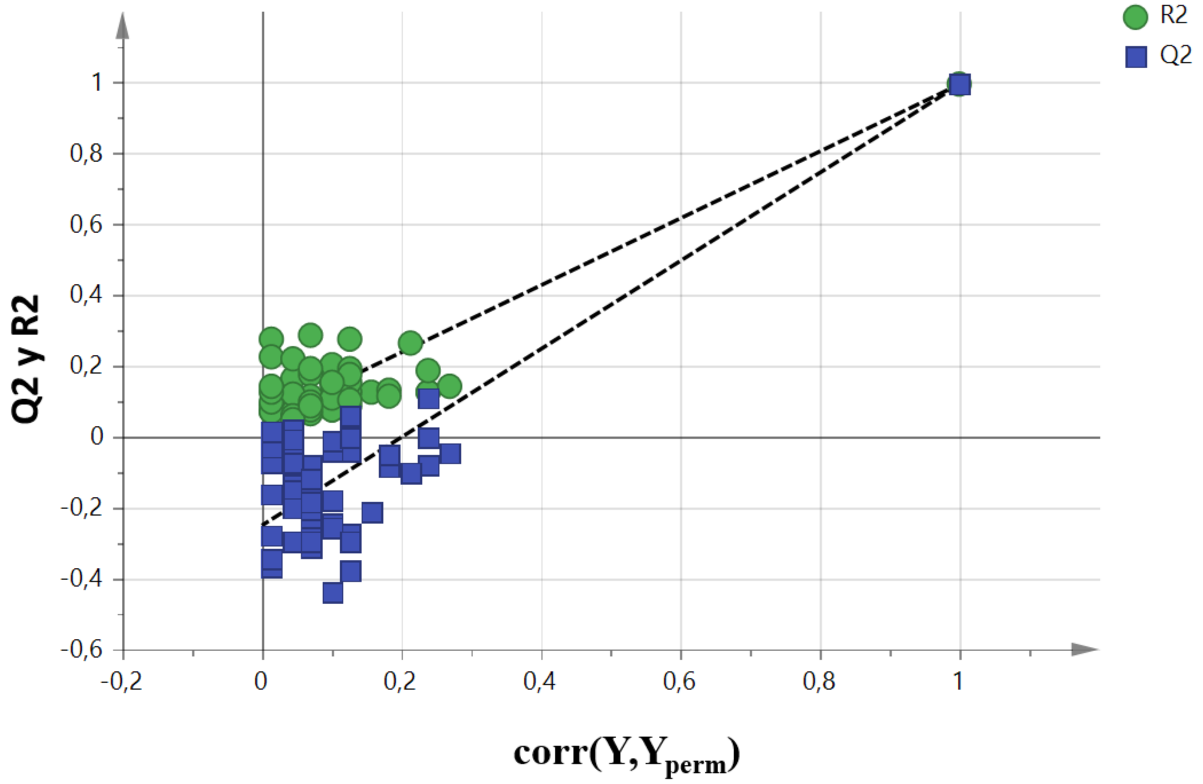

Supplemental Figure S9. Permutation plot for the selected OPLS-DA model. A total of 50 permutations were carried out.

foods MDPI

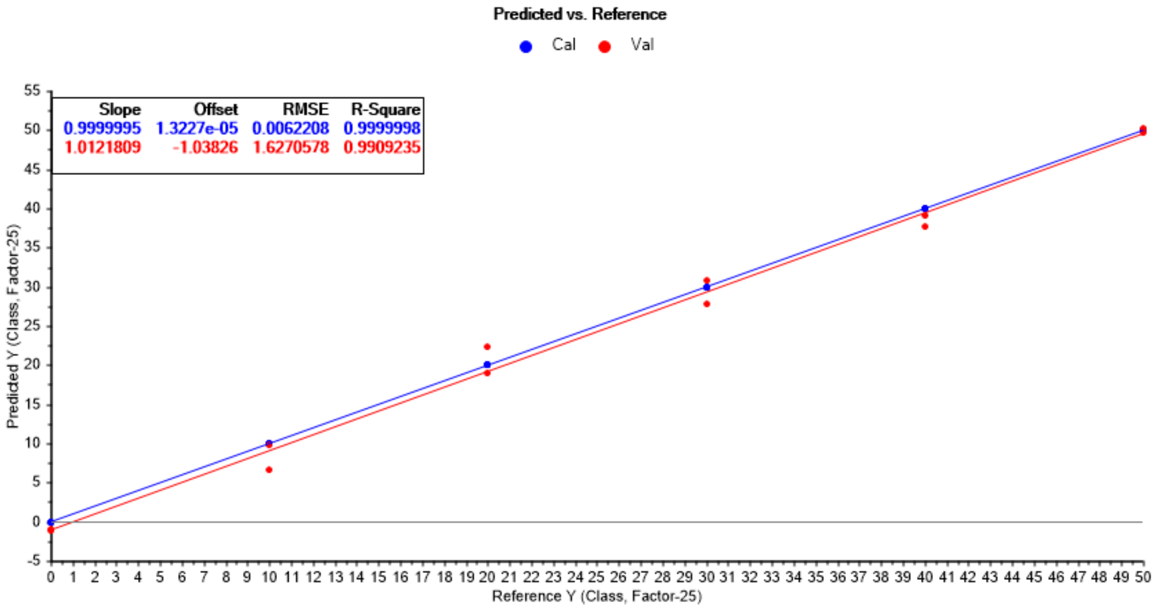

Supplemental Figure S10. PLS regression both with calibration set (blue) and with validation set (red).

foods MDPI

Supplemental Table S1. Water solubility of identified compounds.

Compounds	Water solubility (g/100 mL) at 25 °C[a]	Soluble/Insoluble [e]
2-butanone	22.3	Soluble
Ethyl acetate	8	Soluble
1-penten-3-one	2.19	Soluble
Valeraldehyde	1.17	Soluble
3-methyl-1-butanol	2.67	Soluble
2-methyl-1-butanol	3	Soluble
Trans-2-pentenal	[b]	Insoluble
1-pentanol	2.2	Soluble
Hexanal	0.564 [c]	Soluble
Trans-2-hexen-1-ol	1.6	Soluble
1-hexanol	0.59	Soluble
Cis-2-hexen-1-ol	[b]	Insoluble
Heptanal	0.125	Soluble
Trans-2-heptenal	[b]	Insoluble
Benzaldehyde	0.695	Soluble
Sabinene	0.0002494	Insoluble
1-octen-3-one	0.08954	Insoluble
6-methyl-5-hepten-2-one	0.302	Soluble
2-pentylfuran	0.004184	Insoluble
Octanal	0.056	Insoluble
Trans,trans-2,4-heptanodienal	[b]	Insoluble
p-cymene	0.00234	Insoluble
Limonene	0.000757	Insoluble
Trans-2-octenal	0.06127	Insoluble
γ-terpinene	0.000868 [d]	Insoluble
Terpinolene	0.00095	Insoluble
Linalool	0.159	Soluble
Nonanal	0.0096	Insoluble
Thymol	0.098	Insoluble
Carvacrol	0.125	Soluble

[a] Values obtained in the PubChem database.
[b] Scientific literature talks about insolubility without giving any value.
[c] Solubility at 30 °C.
[d] Solubility at 22 °C.
[e] Compounds with a solubility lower than 0.1 g/100 mL were considered insoluble [38].

foods MDPI

Supplemental Table S2. VOCs identified in ecological Mediterranean oregano and olive leaves (manzanilla or cornicabra).

Compounds	Retention Time (s)	Monomer Drift Time (ms)	Dimer Drift Time (ms)	Present in:
Ethanol/ Oleuropein [a]	223.740	7.605	8.217	Oregano, manzanilla, cornicabra
2-butanone	273.240	7.735	9.291	Oregano, manzanilla
Ethyl acetate	283.140	8.042	9.997	Oregano, manzanilla
1-penten-3-one	339.570	7.746	9.793	Oregano, manzanilla, cornicabra
Valeraldehyde	349.47	8.638	10.623	Oregano, manzanilla, cornicabra
3-methyl-1-butanol	388.08	9.062	11.097	Oregano, manzanilla, cornicabra
2-methyl-1-butanol	391.050	8.956	10.944	Oregano, manzanilla, cornicabra
Trans-2-pentenal	411.840	8.129	10.177	Oregano, manzanilla, cornicabra
1-pentanol	423.72	9.149	11.209	Oregano, manzanilla
Hexanal	460.350	9.209	11.700	Oregano, manzanilla, cornicabra
Trans-2-hexen-1-ol	524.700	8.689	11.379	Oregano, manzanilla, cornicabra
1-hexanol	540.540	9.689	12.216	Cornicabra
Cis-2-hexen-1-ol	541.530	7.499	---	Cornicabra, manzanilla
Heptanal	577.170	9.766	12.729	Oregano, manzanilla, cornicabra
Trans-2-heptenal	638.550	9.254	12.535	Manzanilla
Benzaldehyde	646.470	8.449	11.000	Oregano, manzanilla, cornicabra
Sabinene	659.340	9.059	---	Oregano
1-octen-3-one	660.330	9.197	12.670	Oregano
6-methyl-5-hepten-2-one	668.250	8.772	---	Oregano, manzanilla, cornicabra
2-pentylfuran	674.190	9.315	---	Manzanilla
Octanal	685.080	10.333	13.734	Cornicabra, manzanilla
Trans,trans-2,4-heptanodienal	694.980	8.675	12.158	Manzanilla
p-cymene	710.820	9.039	---	Oregano
Limonene	713.790	9.070	9.597	Oregano
Trans-2-octenal	740.520	9.856	13.712	Manzanilla
γ-terpinene	743.490	9.060	---	Oregano, manzanilla
Terpinolene	774.80	8.842	9.072	Oregano
Linalool	784.080	9.065	---	Oregano
Nonanal	788.040	10.869	14.638	Cornicabra, manzanilla
Thymol	1094.940	9.361	---	[b]
Carvacrol	1121.670	9.405	---	Oregano

[a] Their signals were ruled out due to lack of selectivity.

[b] Thymol was present neither in ecological oregano neither in olive leaves, but, after carvacrol, it is the most important oregano essential oil. It will be present in commercial oregano samples (section 3.6).

foods

Supplemental Table S3. Precision study with identified compounds (RSD, %).

Compounds	Repeatability (n=5)		Intermediate precision (n=3)	
	**1 μg g⁻¹**	**5 μg g⁻¹**	**1 μg g⁻¹**	**5 μg g⁻¹**
2-butanone	3.1	1.6	9.0	4.6
Ethyl acetate	2.2	1.2	6.3	3.8
1-penten-3-one	2.3	0.5	3.0	1.8
Valeraldehyde	1.7	1.5	3.9	3.3
3-methyl-1-butanol	2.8	2.0	10.4	6.9
2-methyl-1-butanol	9.1	1.9	9.1	2.1
Trans-2-pentenal	1.9	0.6	3.7	1.7
1-pentanol	2.8	1.2	9.8	6.6
Hexanal	1.6	1.0	4.9	2.6
Trans-2-hexen-1-ol	7.2	1.5	13.3	2.8
1-hexanol	---	7.1	---	9.6
Cis-2-hexen-1-ol	4.8	1.1	4.9	1.5
Heptanal	2.3	1.7	4.3	3.7
Trans-2-heptenal	4.7	0.8	7.1	4.7
Benzaldehyde	7.9	1.9	11.3	5.9
Sabinene	3.2	2.5	3.6	2.5
1-octen-3-one	2.2	1.8	7.5	2.5
6-methyl-5-hepten-2-one	3.1	1.5	5.2	3.6
2-pentylfuran	5.0	3.9	8.9	7.5
Octanal	---	2.2	---	12.1
Trans,trans-2,4-heptanodienal	---	1.2	---	4.6
p-cymene	---	6.0	---	13.3
Limonene	6.8	4.6	8.3	5.8
Trans-2-octenal	---	2.6	---	13.0
γ-terpinene	---	3.4	---	3.5
Terpinolene	---	3.4	---	4.5
Linalool	---	11.6	---	12.5
	**7.5 μg g⁻¹**	**15 μg g⁻¹**	**7.5 μg g⁻¹**	**15 μg g⁻¹**
Nonanal	6.2	7.1	8.7	9.1
	**2500 μg g⁻¹**	**5000 μg g⁻¹**	**2500 μg g⁻¹**	**5000 μg g⁻¹**
Thymol	4.4	3.0	6.2	3.0
	**5000 μg g⁻¹**		**5000 μg g⁻¹**	
Carvacrol	3.9		4.0	

foods

MDPI

Supplemental Table S4. R^2 of calibration curves obtained with logarithmic regression adjustment.

Compounds	Monomer	Dimer	Monomer and Dimer Sum
2-butanone	0.915	0.984	0.953
Ethyl acetate	0.898	0.987	0.964
1-penten-3-one	0.911	0.991	0.969
Valeraldehyde	0.921	0.952	0.976
3-methyl-1-butanol	0.973	---	---
2-methyl-1-butanol	0.960	---	---
Trans-2-pentenal	0.987	0.977	0.947
1-pentanol	0.985	0.996	0.995
Hexanal	0.945	0.895	0.915
Trans-2-hexen-1-ol	0.967	0.975	0.964
1-hexanol	0.941	0.974	0.976
Cis-2-hexen-1-ol	0.993	---	---
Heptanal	0.987	0.978	0.936
Trans-2-heptenal	0.982	0.953	0.974
Benzaldehyde	0.989	0.980	0.982
Sabinene	0.986	---	---
1-octen-3-one	0.982	0.947	0.975
6-methyl-5-hepten-2-one	0.973	---	---
2-pentylfuran	0.975	---	---
Octanal	0.771	0.991	0.958
Trans,trans-2,4-heptanodienal	0.975	0.961	0.977
p-cymene	0.954	---	---
Limonene	0.862	0.976	0.849
Trans-2-octenal	0.820	0.966	0.974
γ-terpinene	0.989	---	---
Terpinolene	0.976	0.983	0.971
Linalool	0.987	---	---
Nonanal	0.950	---	---
Thymol	0.973	---	---
Carvacrol	0.983	---	---

foods MDPI

Supplemental Table S5. R^2 of calibration curves obtained with the adjustment to Boltzmann´s equation.

Compounds	Monomer	Dimer	Monomer and Dimer Sum
2-butanone	0.9872	0.9986	0.9984
Ethyl acetate	0.9834	0.9976	0.9989
1-penten-3-one	0.9891	0.9994	0.9996
Valeraldehyde	0.9826	0.9989	0.9983
3-methyl-1-butanol	0.9928	---	---
2-methyl-1-butanol	0.9898	---	---
Trans-2-pentenal	0.9960	0.9993	0.9996
1-pentanol	0.9918	0.9986	0.9977
Hexanal	0.9877	0.9980	0.9969
Trans-2-hexen-1-ol	0.9720	0.9957	0.9960
1-hexanol	0.9431	0.9738	0.9764
Cis-2-hexen-1-ol	0.9991	---	---
Heptanal	0.9938	0.9970	0.9976
Trans-2-heptenal	0.9863	0.9992	0.9982
Benzaldehyde	0.9919	0.9959	0.9969
Sabinene	0.9981	---	---
1-octen-3-one	0.9862	0.9981	0.9978
6-methyl-5-hepten-2-one	0.9994	---	---
2-pentylfuran	0.9977	---	---
Octanal	0.8190	0.9910	0.9646
Trans,trans-2,4-heptanodienal	0.9756	0.9957	0.9943
p-cymene	0.9539	---	---
Limonene	0.9974	0.9798	0.9978
Trans-2-octenal	0.8464	0.9916	0.9801
γ-terpinene	0.9914	---	---
Terpinolene	0.9868	0.9874	0.9936
Linalool	0.9924	---	---
Nonanal	0.9928	---	---
Thymol	0.9741	---	---
Carvacrol	0.9985	---	---

foods

MDPI

Supplemental Table S6. Constants of the calibration curves obtaining with the adjustment of the sum of the intensities of protonated monomer and proton-bound dimer to Boltzmann´s equation.

Compounds	A1	A2	dx	x0
2-butanone	-1.27E-02	8.34E-01	1.04E+00	1.10E+00
Ethyl acetate	-1.91E-02	9.28E-01	1.17E+00	1.11E+00
1-penten-3-one	-3.52E-02	9.43E-01	1.21E+00	1.10E+00
Valeraldehyde	1.26E-04	4.47E-01	1.13E+00	7.38E-01
3-methyl-1-butanol	2.08E-03	4.17E-02	7.24E-01	3.46E-01
2-methyl-1-butanol	-1.63E-03	2.60E-02	7.63E-01	8.84E-02
Trans-2-pentenal	-3.67E-02	1.13E+00	1.13E+00	2.53E+00
1-pentanol	-3.70E-02	1.52E-01	1.13E+00	4.77E-01
Hexanal	3.93E-03	4.11E-01	1.10E+00	1.82E+00
Trans-2-hexen-1-ol	-1.11E-02	2.60E-01	8.61E-01	2.23E+00
1-hexanol	-1.23E-02	7.65E-02	7.29E-01	1.62E+00
Cis-2-hexen-1-ol	-3.57E-02	2.77E-01	2.88E+00	4.86E+00
Heptanal	-1.36E-02	4.36E-01	1.17E+00	1.62E+00
Trans-2-heptenal	-2.42E-02	5.64E-01	1.37E+00	2.77E+00
Benzaldehyde	-1.62E-02	2.42E-01	9.72E-01	1.57E+00
Sabinene	-2.62E-02	3.59E-01	1.49E+00	2.27E+00
1-octen-3-one	-4.88E-02	1.17E+00	2.02E+00	5.39E+00
6-methyl-5-hepten-2-one	-3.16E-02	9.79E-01	2.11E+00	5.91E+00
2-pentylfuran	-2.14E-02	3.52E-01	9.85E-01	1.86E+00
Octanal	5.82E-05	7.51E-02	6.19E-01	1.24E+00
Trans,trans-2,4-heptanodienal	-2.41E-02	2.77E+02	1.50E+00	1.40E+01
p-cymene	-6.77E+00	1.92E+00	6.08E+01	-7.59E+01
Limonene	-8.53E-04	2.28E-01	8.32E-01	1.95E+00
Trans-2-octenal	-5.98E-02	1.62E+02	2.49E+00	2.01E+01
γ-terpinene	-7.01E-02	5.19E-01	1.68E+00	3.21E+00
Terpinolene	-1.04E-02	1.95E-01	6.82E-01	3.30E+00
Linalool	-9.87E-03	1.46E-01	8.16E-01	3.26E+00
Nonanal	1.21E-03	5.95E-02	4.85E-01	2.97E+00
Thymol	-1.84E+02	8.96E-01	1.18E+01	-5.63E+01
Carvacrol	-2.14E-01	2.21E+00	3.27E+00	1.43E+01

foods MDPI

Supplemental Table S7. LOD and LOQ of the developed method.

Compounds	LOD (µg g-1)	LOQ (µg g-1)
2-butanone	0.03	0.10
Ethyl acetate	0.03	0.10
1-penten-3-one	0.02	0.08
Valeraldehyde	0.03	0.09
3-methyl-1-butanol	0.14	0.47
2-methyl-1-butanol	0.15	0.51
Trans-2-pentenal	0.03	0.10
1-pentanol	0.15	0.50
Hexanal	0.03	0.09
Trans-2-hexen-1-ol	0.29	0.96
1-hexanol	0.72	2.39
Cis-2-hexen-1-ol	0.14	0.47
Heptanal	0.03	0.10
Trans-2-heptenal	0.12	0.41
Benzaldehyde	0.14	0.48
Sabinene	0.09	0.31
1-octen-3-one	0.14	0.48
6-methyl-5-hepten-2-one	0.14	0.48
2-pentylfuran	0.14	0.47
Octanal	0.69	2.29
Trans,trans-2,4-heptanodienal	0.65	2.15
p-cymene	0.71	2.37
Limonene	0.03	0.10
Trans-2-octenal	0.74	2.45
γ-terpinene	0.66	2.21
Terpinolene	1.44	4.79
Linalool	1.51	5.03
Nonanal	1.35	4.49
Thymol	298	994
Carvacrol	287	956

foods MDPI

Supplemental Table S8. Information about OPLS-DA models.

**Information about models**							**Classification success rate**	
Model	Transformation	Variables	Components	R2X	R2Y	Q2	Calibration	Validation
1	Logarithmic	449	1+1+0	0.613	0.991	0.989	100	100
**2***	**Logarithmic**	**114**	**1+1+0**	**0.846**	**0.994**	**0.994**	**100**	**100**

* Selected model.

Supplemental Table S9. Information about PLS model.

**Correlation**		**Slope**		**R^2**		**RMSE**		**SE**		**Bias**	
Cal	Val	Cal	Val	Cal	Val	RMSEC	RMSEP	SEC	SEP	Cal	Val
0.9999999	0.997	0.999995	1.012	0.9999998	0.9909	6.22E-2	1.63	6.29E-2	1.52	0	-0.73

Cal: calibration set; Val: validation set

R^2: coefficient of determination; RMSEC: root-mean-square error of calibration; RMSEP: root-mean-square error of prediction; SEC: standard error of calibration; SEP: standard error of prediction

foods

MDPI

Supplemental Table S10. Validation of PLS model.

Real percentage of olive leaves (%)	Predicted percentage of olive leaves (%)
0	-1.1±1.1
0	-1.0±1.4
10	9.9±1.5
10	7±2
20	22.4±1.6
20	19.0±1.4
30	27.8±1.2
30	30.8±0.9
40	37.7±0.9
40	39.1±1.4
50	50.2±1.1
50	49.7±1.4

Supplemental Table S11. Quantification of VOCs in commercial oregano samples (μg g^{-1}).

Compounds	Sample 1	Sample 2	Sample 3	Sample 4	Sample 5	Sample 6	Sample 7	Sample 8	Sample 9	Sample 10	Sample 11	Sample 12	Sample 13	Sample 14	Sample 15
2-butanone	0.110	NQ	0.212	NQ	NQ	0.202	0.135	0.159	NQ	NQ	NQ	0.103	NQ	0.706	0.212
Ethyl acetate	ND	NQ	NQ	NQ	NQ	ND	ND	ND	ND	NQ	ND	NQ	ND	ND	ND
1-penten-3-one	0.083	NQ	0.081	0.094	NQ	NQ	NQ	NQ	0.096	NQ	NQ	NQ	NQ	NQ	NQ
Valeraldehyde	0.475	0.511	0.406	0.311	0.772	0.927	1.00	0.546	0.839	0.800	0.815	1.11	0.471	0.617	0.348
3-methyl-1-butanol	ND	NQ	ND	ND	NQ	ND	ND	ND	ND	ND	ND	NQ	ND	NQ	NQ
2-methyl-1-butanol	ND	ND	ND	ND	NQ	ND	ND	ND	ND	ND	ND	NQ	ND	ND	NQ
Trans-2-pentenal	0.708	0.530	0.593	0.612	0.583	0.320	0.359	0.302	0.534	0.320	0.422	0.408	0.355	0.459	0.377
1-pentanol	NQ	NQ	NQ	NQ	NQ	NQ	ND	ND	ND	NQ	ND	NQ	NQ	NQ	NQ
Hexanal	7.83	2.22	2.84	1.86	2.36	1.69	1.29	1.54	1.70	1.55	1.29	1.18	0.631	0.932	0.450
Trans-2-hexen-1-ol	5.26	4.82	6.07	>10	7.89	2.91	4.24	2.85	>10	2.82	>10	6.93	>10	7.84	6.32
1-hexanol	ND	ND	ND	ND	ND	NQ	ND	ND	ND	ND	ND	ND	ND	ND	ND
Cis-2-hexen-1-ol	ND	NQ	ND	ND	NQ	NQ	ND	NQ	ND	ND	ND	NQ	NQ	NQ	NQ
Heptanal	0.206	0.122	0.126	0.109	0.113	NQ	NQ	0.113	NQ	NQ	NQ	ND	NQ	NQ	ND
Trans-2-heptenal	0.535	NQ	NQ	NQ	ND	NQ	0.475	NQ	NQ	NQ	NQ	NQ	NQ	NQ	ND
Benzaldehyde	0.634	1.569	0.646	0.551	0.671	0.646	0.574	0.598	0.745	NQ	NQ	0.783	NQ	0.745	0.770

1-octen-3-one	2.28	2.17	2.09	2.15	2.22	ND	ND	ND	2.75	ND	2.85	2.56	2.03	2.07	1.90
6-methyl-5-hepten-2-one	0.517	NQ	NQ	NQ	NQ	NQ	NQ	0.586	NQ	NQ	NQ	ND	NQ	NQ	ND
2-pentylfuran	ND	ND	ND	ND	ND	ND	ND	ND	ND	ND	ND	ND	ND	ND	ND
Octanal	NQ	ND	NQ	ND	ND	ND	ND	ND	ND	ND	ND	ND	NQ	ND	NQ
Trans,trans-2,4-heptanodienal	NQ	ND	NQ	NQ	ND	ND	ND	ND	ND	ND	ND	ND	ND	ND	ND
Limonene	>10	>10	>10	>10	>10	>10	>10	9.89	>10	5.85	>10	5.85	>10	>10	>10
Trans-2-octenal	ND	ND	ND	ND	ND	ND	ND	ND	ND	ND	ND	ND	ND	ND	ND
Terpinolene	>10	>10	>10	>10	>10	ND	NQ	ND	>10	ND	>10	>10	>10	>10	>10
Nonanal	ND	ND	ND	ND	ND	ND	ND	ND	ND	ND	ND	ND	ND	ND	ND

ND: not detected, NQ: detected, but not quantified
